# ntHash2: recursive spaced seed hashing for nucleotide sequences

**DOI:** 10.1093/bioinformatics/btac564

**Published:** 2022-08-24

**Authors:** Parham Kazemi, Johnathan Wong, Vladimir Nikolić, Hamid Mohamadi, René L Warren, Inanç Birol

**Affiliations:** Canada’s Michael Smith Genome Sciences Centre, British Columbia Cancer Agency, Vancouver, BC V5Z 4S6, Canada; Faculty of Science, University of British Columbia, Vancouver, BC V6T 1Z4, Canada; Canada’s Michael Smith Genome Sciences Centre, British Columbia Cancer Agency, Vancouver, BC V5Z 4S6, Canada; Canada’s Michael Smith Genome Sciences Centre, British Columbia Cancer Agency, Vancouver, BC V5Z 4S6, Canada; Amazon Web Services Inc., Seattle, WA 98109, USA; Canada’s Michael Smith Genome Sciences Centre, British Columbia Cancer Agency, Vancouver, BC V5Z 4S6, Canada; Canada’s Michael Smith Genome Sciences Centre, British Columbia Cancer Agency, Vancouver, BC V5Z 4S6, Canada; Department of Medical Genetics, University of British Columbia, Vancouver, BC V6T 1Z3, Canada

## Abstract

**Motivation:**

Spaced seeds are robust alternatives to *k*-mers in analyzing nucleotide sequences with high base mismatch rates. Hashing is also crucial for efficiently storing abundant sequence data. Here, we introduce ntHash2, a fast algorithm for spaced seed hashing that can be integrated into various bioinformatics tools for efficient sequence analysis with applications in genome research.

**Results:**

ntHash2 is up to 2.1× faster at hashing various spaced seeds than the previous version and 3.8× faster than conventional hashing algorithms with naïve adaptation. Additionally, we reduced the collision rate of ntHash for longer *k*-mer lengths and improved the uniformity of the hash distribution by modifying the canonical hashing mechanism.

**Availability and implementation:**

ntHash2 is freely available online at github.com/bcgsc/ntHash under an MIT license.

**Supplementary information:**

Supplementary data are available at *Bioinformatics* online.

## 1 Introduction

Many applications in bioinformatics utilize hashing algorithms to efficiently populate and query various data structures. Previously, we introduced ntHash (Mohamadi *et al.*, 2016), a recursive function for hashing consecutive substrings of length k (k-mers) in nucleotide sequences.

With the advent of high-throughput sequencing and rise in popularity of Illumina, Inc. (San Diego, USA) short-read sequencing, k-mer-based analysis solutions have flourished. Even though they show versatility in normalizing highly accurate sequencing data, k-mers fail to distinguish between similar sequences that may exist due to polymorphisms or arise from sequencing errors. Error tolerance is particularly important today as long reads technologies offered by Oxford Nanopore Technologies PLC (Oxford, UK) or Pacific Biosciences of California, Inc. (Menlo Park, CA, USA) gain in popularity, despite their appreciable error rates. To allow a deterministic level of tolerance for base mismatches, k-mers can be replaced by spaced seeds, i.e. patterns of ‘care’ and ‘do not care’ positions. Spaced seeds are used routinely in sequence analysis applications, such as homology search (Ma *et al.*, 2002) and classification (Chu *et al.*, 2020).

Few algorithms are optimized for spaced seed hashing. Typically, users take the inefficient approach of replacing ‘do not care’ positions with an ignored character and then employ a k-mer hashing algorithm to hash the masked string. In this work, we developed an efficient spaced seed hashing method by leveraging the properties of recursive hashing.

## 2 Methods

In ntHash (Mohamadi *et al.*, 2016), one initially computes a 64-bit hash value for the first k-mer in a longer sequence. Subsequent hash values are then generated by removing the first character of the previous k-mer and including the next character in the sequence using left rotation (rol) and XOR operations (Supplementary Section S1). However, since ${\mathrm{rol}}^{64}\left(x\right)=x$ when storing rotation outputs in 64-bit words, this raises the issue of rotational periodicity and leads to increased hash collision rates for higher values of k. We address this issue in ntHash2 by replacing rol with a new function called srol, short for split rotation. To compute the srol of a 64-bit word x, we first split x into $d_{1},\ldots ,d_{n}$-bit sub-words ($\sum d_{i}=64$ and $\forall i,j:\;\text{gcd}\left(d_{i},d_{j}\right)=1$), rotate the sub-words separately, and finally join the results according to their placement in x. Hence, the period of srol is $\text{lcm}(d_{1},\ldots ,d_{n})$, which makes it more suitable than rol for bioinformatics applications with longer *k*-mer lengths (Supplementary Section S2).

To improve the uniformity of hash distribution, we define the canonical hash value of each seed as the sum of the forward-strand and reverse-complement hashes as a replacement for the minimum of the hashes, which was used in the previous version (Supplementary Section S3).

The main novelty of ntHash2 is our approach to spaced seed hashing. Let s be a spaced seed composed of w 1s (seed weight) and k-w 0s as ‘care’ and ‘do not care’ positions, respectively. We define a *block* as a consecutive run of 1s flanked by 0s or the seed’s ends. First, we store the indices of the first ‘1’ in each block and the first ‘0’ after the block in a list B. Blocks of size one, or *monomers*, are stored and encoded separately to prevent excess XOR operations. To find a hash value for the first |s| characters, we iterate over block ranges and include the characters using srol and XOR operations with time complexity of O(w). We then generate each subsequent hash by removing the character respective to the blocks’ first indices and including the characters pointed by the second indices, taking $O(\left|B\right|)$ time. We finally include the positions stored in the list of monomers, resulting in two XOR operations for each block and one for each monomer. For faster computation, ntHash2 redefines blocks as stretches of 0s if the number of predicted XOR operations is fewer for excluding 0s from the hash value (Supplementary Section S4).

As before, ntHash2 can perform reverse-complement hashing without requiring extra iterations by swapping the corresponding indices in the blocks. Additionally, ntHash2 can generate multiple hashes per spaced seed, roll backwards in the input sequence and process a stream of characters in real-time (Supplementary Section S5).

## 3 Results and discussion

**Fig. 1.**
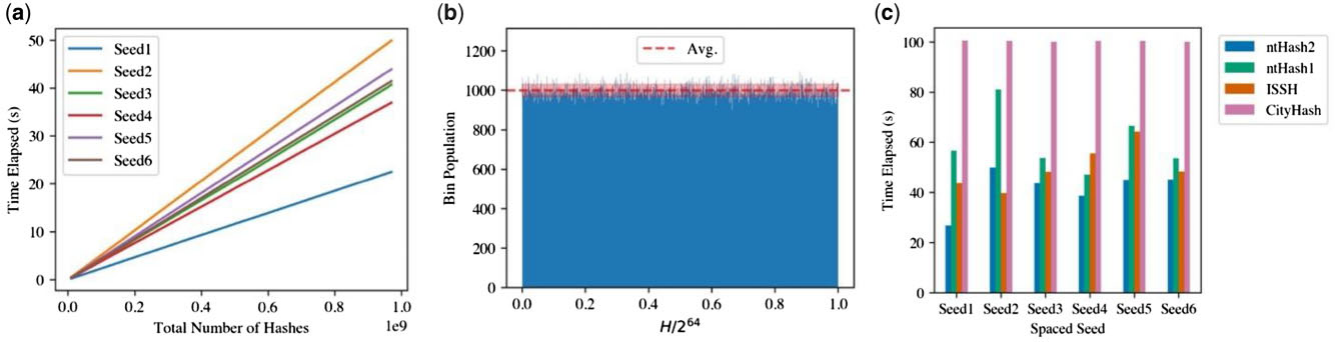
(**a**) Linear increase of the wall clock time required by ntHash2 to generate spaced seed hashes (Seeds 1–6) from 1 million random 1 kbp sequences (one hash value per seed). Hashing spaced seeds with more blocks and monomers takes more time. (**b**) Histogram of a million k-mer hashes generated by ntHash2 from random 100-mers. Hash values (H) are distributed uniformly in the normalized 64-bit word space (*x*-axis). The mean and standard deviation of the bin counts are 1000 ± 31.29, which is close to the ideal value of 1000 hashes per bin. (**c**) Average wall clock time elapsed by ntHash2 and similar hashing algorithms on a unique dataset (10^6^ random 1 kbp sequences) over 3 runs. Standard deviation was negligible (<500 ms for all tools). Spaced seed patterns (Seed1–Seed6) are described in Supplementary Section S6

We evaluate ntHash2 using randomly generated sequences to show its independence from the nature of the data. The spaced seeds used in our experiments showcase various conditions and their impact on ntHash2’s performance (Supplementary Section S6). As expected, run times scale linearly with respect to the number of processed substrings, and hashing seeds with more blocks and monomers takes more time (Fig. 1a).

We note that, uniform hash distribution would reduce the probability of collisions. To show the uniformity of ntHash2, we use the Kolmogorov–Smirnov (K-S) test (Chakravarti *et al.*, 1967). The histogram of 10^6^  k-mer hashes generated by ntHash2 from random data is statistically indistinguishable from a uniform distribution (K-S statistic of 0.0007 and *P*-value of 0.62, Fig. 1b).

Finally, we compare the performance ntHash2 with competing algorithms. The previous version (ntHash1) produces hashes for spaced seeds by removing the ‘do not care’ positions using XORs. CityHash is a general-purpose hash function, which we adapted to spaced seed hashing by replacing the ‘do not care’ positions in each substring with an asterisk. Iterative Spaced Seed Hashing (ISSH) (Petrucci *et al.*, 2020) is a spaced seed hash function that reuses previous hashes based on the seed’s overlapping patterns. Because CityHash and ISSH lack canonical hashing, we also fed the reverse-complement of the input sequences to compare run times with ntHash2. ntHash2 outperforms ntHash1 and CityHash at spaced seed hashing by 1.2–2.1× and 2–3.8×, respectively (Fig. 1c). Compared to ISSH, ntHash2 is up to 1.6× faster, other than seed 2 which has $\left|s\right|/2$ monomers and is one of the less-optimal input seeds for ntHash2.

Overall, ntHash2 is a versatile and scalable spaced seed hashing algorithm for nucleotide sequences with various use cases, such as genome assembly and k-mer counting.

## Funding

This work was supported by the National Institutes of Health [2R01HG007182-04A1]. The content of this work is solely the responsibility of the authors and does not necessarily represent the official views of the National Institutes of Health.

*Conflict of Interest*: none declared.

## Supplementary Material

btac564_Supplementary_Data

## Data Availability

Scripts for generating the random data used in the experiments are available online at github.com/bcgsc/ntHash. Additional data used in the extended experiments (Supplementary Section 7) are available in the supplementary information.
