# Supplementary material for: ntHash2: recursive spaced seed hashing for nucleotide sequences
Source: Bioinformatics. 2022 Aug 24;38(20):4812–3. doi: 10.1093/bioinformatics/btac564 (PMC9563681; doi:10.1093/bioinformatics/btac564)
Supplement: btac564_Supplementary_Data [file btac564_supplementary_data.pdf]

Supplementary material for ntHash2: Recursive Spaced Seed Hashing for Nucleotide Sequences

Parham Kazemi, Johnathan Wong, Vladimir Nikolić, Hamid Mohamadi, René L Warren, and Inanç Birol

Table of Contents

1. **Background** 3
2. **Updated k-mer Hashing** 3
3. **Uniformity of the Canonical Hash Value** 6
4. **Spaced Seeds Hashing Algorithm** 9
5. **Additional Features** 11
6. **Spaced Seeds Used in Experiments** 11
7. **Extended Experiments and Results** 11

References 14

Table of Figures

Fig. S1. Implementation of the $srol$ function. 5

Fig. S2. Implementation of the $sror$ function. 5

Fig. S3. Generating extended hash values for each k-mer. 5

Fig. S4. Fetching pre-computed $srol$ values. 6

Fig. S5. Histogram of the generated hashes for forward and reverse-complement sequences. Normalized hash values are shown on the x-axis. 7

Fig. S6. Probability density function of the Irwin-Hall distribution generated by simulation... 8

Fig. S7. Histograms of canonical hashes generated with min, add, and xor operators. Hi refers to the ith hash value generated from each k-mer. 9

Fig. S8. Pseudocode of the spaced seed hashing procedure. 10

Fig. S9. C. elegans k-mer profiles generated with ntCard using both the previous and new versions of ntHash (k=80). ntCard is also able to estimate k-mer profiles with low error when integrated with ntHash2. 13

Fig. S10. Dataset coverage estimation using ntCard with k-mers and spaced seeds. The plot on the right is a zoomed-in version of the peak in the count range between 34 and 64. Spaced seeds are more stable compared to k-mers with the same number of bases and are more reliable for coverage estimation in long-read datasets. 14

1. Background

Given a sequence r containing characters from an alphabet Σ, ntHash initially computes a base hash value H_0 for the first k-mer. Next, for each succeeding k-mer, the contribution of the previous k-mer's first character is removed and the next character in the sequence is included using circular shifts and exclusive-or (XOR) operations. Formally, ntHash can be shown as a recursive function:

$$H_i = \begin{cases} rol^{k-1}(h[r_0]) \oplus \dots \oplus rol(h[r_{k-2}]) \oplus h[r_{k-1}] & i = 0 \\ rol(H_{i-1}) \oplus rol^k(h[r_{i-1}]) \oplus h[r_{i+k-1}] & i > 0 \end{cases} \quad (1)$$

where $rol^d(x)$ is the result of rotating a 64-bit word d times to the left and h is a lookup table for mapping each character in Σ to a random 64-bit integer. ntHash also calculates the hash value of the reverse-complement of each k-mer in the same loop as the forward strand, which leads to faster canonical hash computation (Mohamadi, Chu, et al. 2016). In our implementation, as $\Sigma = \{A, C, G, T\}$, ntHash performs DNA hashing and ignores any other character not present in this alphabet.

2. Updated k-mer Hashing

In equation (1), since rotating any x 64 times results in the same value, when $k > 64$, swapping two distinct characters 64 positions apart will not alter the resulting base hash value. Also, the rotation terms of two identical characters with a distance of 64 positions cancel each other out, resulting in the same effect. Our solution to this issue is to replace rol with $srol$, which has a higher period. Because modern computers operate on 64-bit words, the period of $srol$ is maximized when $d_1 = 31$ and $d_2 = 33$ ($\mathrm{lcm}(31, 33) = 1023$), where lcm stands for the least common multiple. Although this operation can be further generalized for longer periodicities by increasing the number of sub-words of co-prime lengths (Table S1), we implement $srol$ with two sub-words in ntHash2. The theoretical maximum periodicity of the $srol$ operation using 64-bit words is 2,042,040 with a pattern of seven sub-words.

Table S1. Generalized optimal split patterns for the *srol* function. The maximum number of possible splits for a 64-bit hash value is seven.

# Splits	Period	Split Bits						
-	64	64						
2	1,023	31	33					
3	9,660	20	21	23				
4	62,985	13	15	17	19			
5	306,306	9	11	13	14	17		
6	855,855	5	7	9	11	13	19	
7	2,042,040	3	5	7	8	11	13	17

The hash value for the ith k-mer in the sequence r is computed by ntHash2 according to the recursive function:

$$H_i = \begin{cases} \bigoplus_{pos=0}^{k-1} srol^{k-1-pos}(h[r_{pos}]) & i = 0 \\ srol(H_{i-1}) \oplus srol^{k}(h[r_{i-1}]) \oplus h[r_{i-1+k}] & i > 0 \end{cases} \quad (2)$$

where $\oplus$ is the bitwise XOR operator, h is a lookup table containing a fixed 64-bit word for each of the characters in the alphabet Σ. Also, *srol* is the split-and-rotate function performed by shifting the input to the left and replacing the bits in positions 0 and 33 with the bits in positions 32 and 63 prior to the shift, as implemented in Fig. S1.

ntHash2 is also able to compute a hash value for the reverse-complement of the input k-mer by modifying the recursion as:

$$H'_i = \begin{cases} \bigoplus_{pos=0}^{k-1} srol^{pos}(h[r'_{pos}]) & i = 0 \\ sror(H'_{i-1} \oplus h[r'_{i-1}] \oplus srol^{k}(h[r'_{i-1+k}])) & i > 0 \end{cases} \quad (3)$$

```
inline uint64_t srol(const uint64_t x) {
  uint64_t m = ((x & 0x8000000000000000ULL) >> 30) |
               ((x & 0x100000000ULL) >> 32);
  return ((x << 1) & 0xFFFFFFFDFFFFFFFFULL) | m;
}
```

Fig. S1. Implementation of the *srol* function.

```
inline uint64_t sror(const uint64_t x) {
  uint64_t m = ((x & 0x200000000ULL) << 30) | ((x & 1ULL) << 32);
  return ((x >> 1) & 0xFFFFFFFEFFFFFFFFULL) | m;
}
```

Fig. S2. Implementation of the *sror* function.

where r' is the complementary base pair of r and *sror* is the reverse of a single *srol*, which is implemented as Fig. S2. In other words, $sror\big(srol(x)\big) = x$.

Additionally, ntHash2 can generate extra hash values for each k-mer using the function shown in Fig. S3. Note that k is the k-mer size and $i > 0$ is the index of the newly generated hash value. MULTISEED and MULTISHIFT are constant 64-bit unsigned integers.

```
inline uint64_t nte64(const uint64_t h_val, const unsigned k, const unsigned i) {
  uint64_t t_val = h_val;
  t_val *= (i ^ k * MULTISEED);
  t_val ^= t_val >> MULTISHIFT;
  return t_val;
}
```

Fig. S3. Generating extended hash values for each k-mer.

To reduce the work required to compute $srol^d(x)$ during runtime, we store results of rotating the first 33 and last 31 bits of each $h[c]$ ($c \in \Sigma$) for all possible rotation counts in two separate pre-defined arrays called MS_TAB_33R and MS_TAB_331. The pre-computed value of $srol^d(x)$ is the result of joining the corresponding elements of the arrays using the bitwise OR operator. This functionality is implemented as Fig. S4.

```
inline uint64_t srol(const unsigned char c, const unsigned d) {
  return (MS_TAB_31L[c][d % 31] | MS_TAB_33R[c][d % 33]);
}
```

Fig. S4. Fetching pre-computed $srol$ values.

Finally, we speed up hashing the first k-mer by pre-computing the hash value of every possible m-mer of length $m \leq 4$ in four 4^m arrays. To form H_0, we first iterate over the 4-mers of the first k-mer and join the respective pre-calculations using $srol$ and XOR operations. If k is not divisible by 4, we fetch the value of the last m characters from the array corresponding to $m = k\%64$.

Naturally, split-rotation is a more complex function compared to simple rotation and requires a higher number of instructions in the CPU. Calling $srol$ 10^{10} times requires approximately 9.4 s to execute, whereas the same number of executions takes around 6.9 s for rol. However, the numerous implementation optimizations we explained in this section compensate for the decrease in performance. Calling the forward hash functions in ntHash1 and ntHash2 10^6 times takes 6.3 s and 6.4 s respectively for random 100-mers on average for 3 test repeats. In the end, the uniformity (Section 3) and room for length increasing that split-rotation brings to ntHash2 is valuable for bioinformatics applications and the slight performance change is negligible in most cases.

3. Uniformity of the Canonical Hash Value

To obtain a single representation for the forward and the reverse complement hash value of k-mers, we define a canonical hash value. This feature is most useful in k-mer counting algorithms. In most bioinformatics applications, the lexicographically smallest between the forward and reverse-complement is selected as the canonical k-mer. Accordingly, the canonical hash value is defined as the minimum of the hashes computed for the forward and reverse-complement of each k-mer in ntHash (Mohamadi, Chu, et al. 2016).

While ntHash was shown to distribute output values uniformly in the 64-bit hash space (Birol, Mohamadi and Chu 2018), we changed the canonical hashing mechanism in ntHash2 to improve its performance and uniformity. Specifically, we compute the canonical hash value as the sum of the hashes generated for the k-mer's forward and reverse-complement. Here, we provide mathematical justification for the selection of addition for canonical hashing.

First, we show that the hash value of the forward and reverse-complements of the sequences are independently uniform. This was shown to be true in ntHash for the rol operator (Birol,

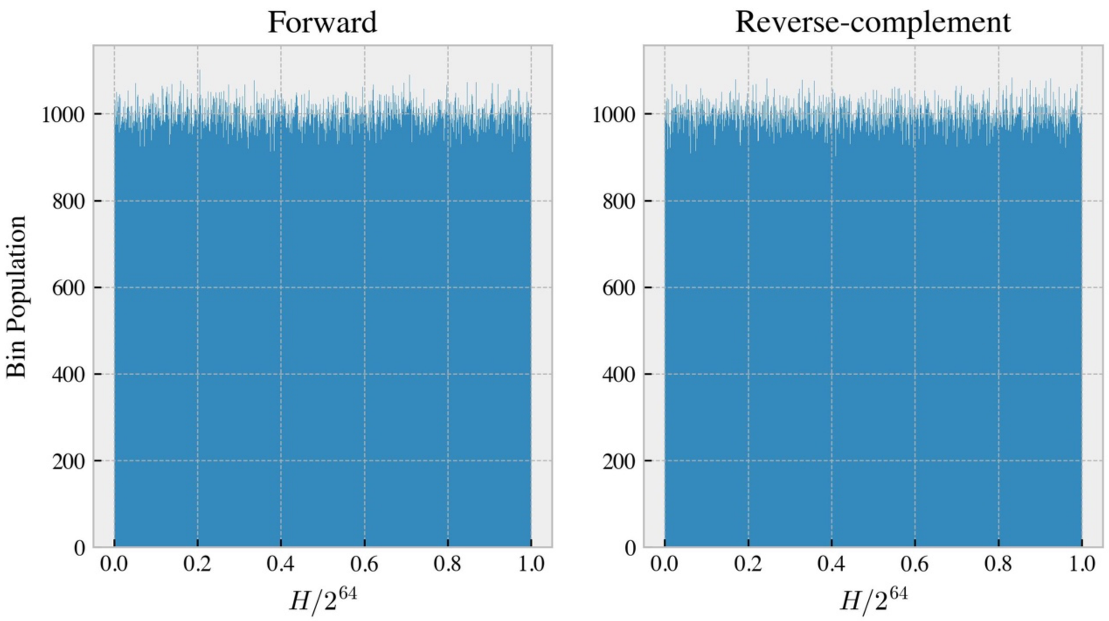

Fig. S5. Histogram of the generated hashes for forward and reverse-complement sequences. Normalized hash values are shown on the x-axis.

Mohamadi and Chu 2018). To do so for ntHash2 and $srol$, we generate 10^6 random k-mers ($k = 100$ for this test) and plot the histogram of the generated hashes by counting the values separated in 1000 bins. As illustrated in Fig. S5, both the forward and reverse-complement hash function are uniformly distributed ($U(0,1)$).

Moreover, we perform the Kolmogorov–Smirnov (K-S) test (Massey Jr. 1951) to compare the distribution of the normalized hash values with $U(0,1)$. According to the test statistics $D = 0.0009$ and $D = 0.0008$ and p-values of $p = 0.48$ and $p = 0.39$ for the forward and reverse strand hashes respectively, we fail to reject the null hypothesis that the distributions are not uniform. Therefore, the distributions of the hashes generated for the forward and reverse-complements of the k-mers are statistically indistinguishable from $U(0,1)$.

After observing a uniform distribution for the output hash values, we prove that in the case of ntHash2, the summation of two uniform distributions results in another uniform distribution. The probability density function (PDF) of the sum of n independent $U(0,1)$ random variables is equal to the Irwin-Hall distribution (Johnson, Kotz and Balakrishnan 1995):

$$f_X(x) = \frac{1}{2(n-1)!}\sum_{k=0}^{n}(-1)^k\binom{n}{k}(x-k)^{n-1}\mathrm{sgn}(x-k) \tag{4}$$

where sgn(·) is the sign function. The PDF is shown in Fig. S6. Note that based on the central limit theorem, the Irwin-Hall distribution estimates a normal distribution as n increases.

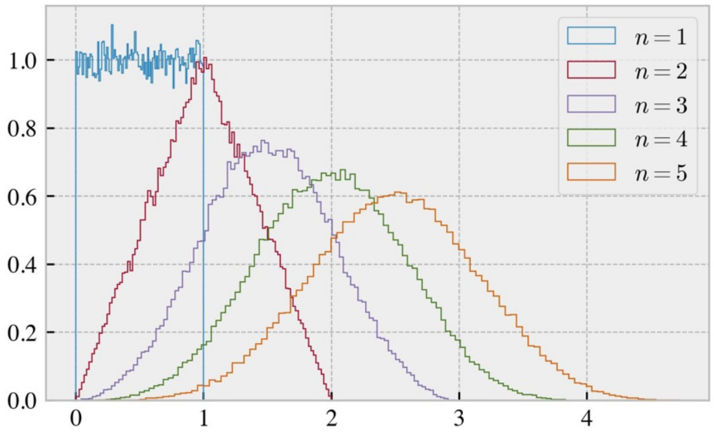

Fig. S6. Probability density function of the Irwin-Hall distribution generated by simulation.

In ntHash2, we have a special case of the Irwin-Hall distribution where $n = 2$:

$$f_X(x) = \begin{cases} x & 0 \leq x < 1 \\ 2 - x & 1 \leq x < 2 \end{cases} \tag{5}$$

According to the fact that ntHash2 operates in the 64-bit unsigned integer space, if the canonical hash value is greater than 2^{64}, integer overflow causes the values to wrap around. The summation of two 64-bit integer values X and Y is simply performed as $mod(X + Y, 2^{64})$ in many computer architectures, and if not, we ensure that this is how it is performed. In that case, the line segment $f_X(x) = 2 - x$ in Fig. S6 for $n = 2$ is transferred to $0 \leq x < 1$ and the superposition of the two line segments creates a uniform distribution.

Formally, the wrap around version of the normalized operation can be formulated as

$$x' = \begin{cases} x & 0 \leq x < 1 \\ x - 1 & 1 \leq x < 2 \end{cases} \tag{6}$$

Because the two cases in x' are mutually exclusive, its probability distribution can be written as

$$f_{X'}(x') = f_X(x') + f_X(x' + 1) = 1 \tag{7}$$

defining a uniform distribution.

Hence, the probability distribution of the canonical hash value defined as the summation of the forward and reverse hashes is uniform.

Other canonical hashing operators tested in previous releases of ntHash are shown in Fig. S7. We also tested employing extended hashes in various applications. It is observed that *min* without extended hash generation is the only operator that is non-uniform. To make ntHash2 uniform when using *min* as the canonical hash operator, a second hash value must be generated which increases

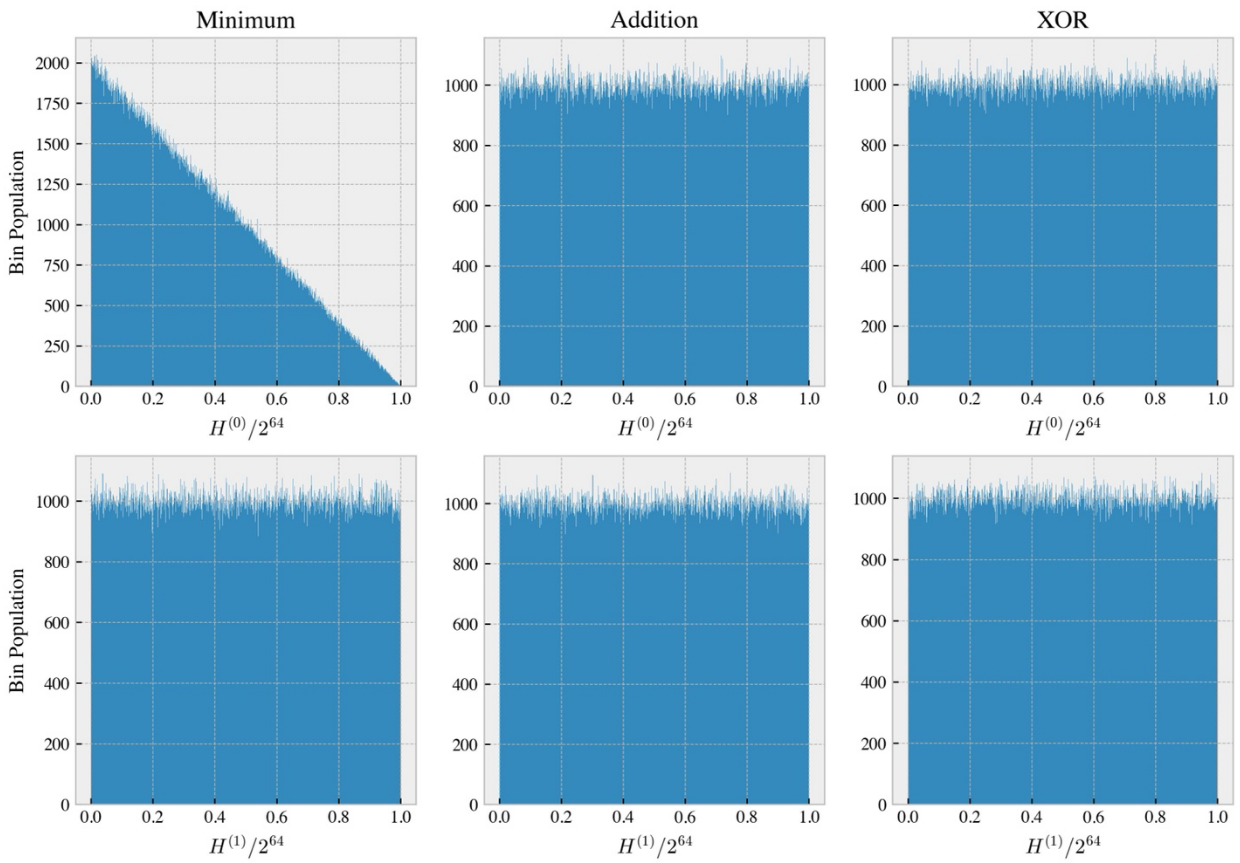

Fig. S7. Histograms of canonical hashes generated with min, add, and xor operators. $H^{(i)}$ refers to the ith hash value generated from each k-mer.

hashing time. On the other and, using XOR for generating canonical hash values results in higher hash collision rates, as the computation of forward and reverse hash values already consist of XOR operations between the characters, and XORing the polynomials of the strands will cause multiple terms to be cancelled out. By using addition as the canonical hash operator in ntHash2, we benefit from fast computation, low collision rates, and high uniformity without the need to generate extra hashes.

4. Spaced Seeds Hashing Algorithm

We summarize our hashing algorithm in the pseudocode presented in Fig. S8. The first stage of our hashing algorithm consists of parsing the input seed strings to blocks and monomers, as implemented in parse_seed. Note that we repeat the same process for blocks of do not cares and use the '0' blocks as masks if the predicted workload is less than blocks of care positions. We

```
function parse_seed(seed_string):
    blocks, monomers, start, is_block = [], [], 0, true
    for i = 0 → seed_string.length():
        if seed_string[i].is_care() and not is_block:
            is_block = true
            start = i
        else if not seed_string[i].is_care() and is_block:
            if i− start > 1:
                add <start, i> to blocks
            else:
                add i to monomers
            is_block = false
    return blocks, monomers

function base_hash(sequence, k, blocks, monomers):          // k is the spaced seed's length
    hash_results = {H, H′, H_b, H_b′}
    for p, q ∈ blocks:
        for i = p → q:
            H = H ⊕ srol(sequence[i], k − i − 1)
            H′ = H′ ⊕ srol(sequence[i]′, i)
        H_b, H_b′ = H, H′
        for i ∈ monomers:
            H = H ⊕ srol(sequence[i], k − i − 1)
            H′ = H′ ⊕ srol(sequence[i]′, i)
    return hash_results

function slide_hash(sequence, k, blocks, monomers, *hash_results):
    H_b = srol(H_b)
    for p, q ∈ blocks:
        H_b = H_b ⊕ srol(sequence[p], k − p) ⊕ srol(sequence[q], k − q)
        H_b′ = H_b′ ⊕ srol(sequence[p]′, p) ⊕ srol(sequence[q]′, q)
    H_b′ = sror(H_b′)
    H, H′ = H_b, H_b′
    for i ∈ monomers:
        H = H ⊕ srol(sequence[i], k − i − 1)
        H′ = H′ ⊕ srol(sequence[i]′, i)
```

Fig. S8. Pseudocode of the spaced seed hashing procedure.

generate a hash value for the first k-mer with base_hash. Forward and reverse hash values before encoding the monomers are stored in H_b and $H_b{}'$ respectively for future function calls. Finally,

subsequent hashes are computed using the slide_hash function.

5. Additional Features

Here, we provide a list of new features included in ntHash2:

- **Streaming:** Users can feed input characters to ntHash2 during runtime via the BlindNtHash class. Only the first k-mer needs to be given to the constructor. Following hash values are generated by passing the next character to the roll(char_in) function.
- **Rolling Back:** NtHash classes provide a roll_back function that reverts the previous roll operation. Bi-directional traversal is possible using this function.

6. Spaced Seeds Used in Experiments

The seeds used in experiments of the main text are provided in Table S2. Seeds 1 and 2 show the upper and lower bounds for the number of blocks and monomers. Seed 3 is designed for maximizing the hit probability (Ounit and Lonardi 2016). Seeds 4 and 5 are randomly generated strings with an average number of blocks and monomers. Seed 6 is designed to maximize the sensitivity (Hahn, et al. 2016).

Table S2. Seed set for the main text's experiments.

Seed1	1111111111000000000011111111111
Seed2	1010101010101010101010101010101
Seed3	1111011101110010111001011011111
Seed4	1111101111101000111111011110011
Seed5	1111000111111010010101010100111
Seed6	1111110101101011100111011001111

7. Extended Experiments and Results

To show that ntHash2 works well when used in tools that rely on k-mer or spaced seed hashing, we integrated it in our *de novo* genome assembler ABySS 2.4.3 (Jackman, et al. 2017) and assembled data from the N2 strain of *C. elegans* (SRA: DRR008444) and the human individual NA24385, i.e. the Ashkenazi son (SRA: SRR11321732) from the Genome in a Bottle project

(Zook, et al. 2016), with ~58x coverage short read data. ABySS parameters are presented in Table S4. Evaluation results are obtained using QUAST (Gurevich, et al. 2013). Tables S3 and S4 show that using ntHash2 as a substitute for the previous version of ntHash does not negatively impact assembly quality. We observe that improved uniformity does not lead to a significant increase in assembly quality in this experiment due to the fact that ntHash2 operates on a very low level of the assembly algorithm.

Table S3. Assembly evaluation results for *C. elegans* data.

ABySS Hash Function	N50 length (bp)	NG50 length (bp)	NGA50 length (bp)	Number of Misassemblies	Number of Local Misassemblies	Genome Fraction	Total Length
ntHash	35,475	33,494	31,208	344	186	95.06%	96,102,680
ntHash2	35,452	33,470	31,218	342	186	95.06%	96,102,377

Table S4. Assembly evaluation results for *H. sapiens* NA24385 data.

ABySS Hash Function	N50 length (bp)	NG50 length (bp)	NGA50 length (bp)	Number of Misassemblies	Number of Local Misassemblies	Genome Fraction	Total Length
ntHash	123,763	106,978	103,290	1,441	1,015	92.53%	2,742,575,128
ntHash2	123,716	106,810	103,255	1,439	1,014	92.53%	2,742,543,401

Table S5. ABySS parameters for the assembly experiments.

Dataset	k	kc	l	s	q	B	H	S	N
C. elegans	80	2	40	1000	15	5G	4	1000-10000	15
H. sapiens NA24385	112	3	40	1000	15	100G	4	1000-10000	9

We also used the hashes generated by ntHash2 in our k-mer counting tool, ntCard (Mohamadi, Khan and Birol, ntCard: a streaming algorithm for cardinality estimation in genomics data 2017) on the same *C. elegans* dataset in the assembly experiment. Results with hashes generated with ntHash2 and ntHash1 are compared with the gold standard, DSK (Rizk, Lavenier and Chikhi 2013), in Fig. S9, where it is observed that using ntHash2 with ntCard results in similarly accurate

profile estimations as of ntHash (0.021% absolute error vs. DSK on average). Again, although ntHash2 is a more uniform hash funcation than ntHash1, some applications such as ntCard may not show an immediate quality gain for all datasets and experiments.

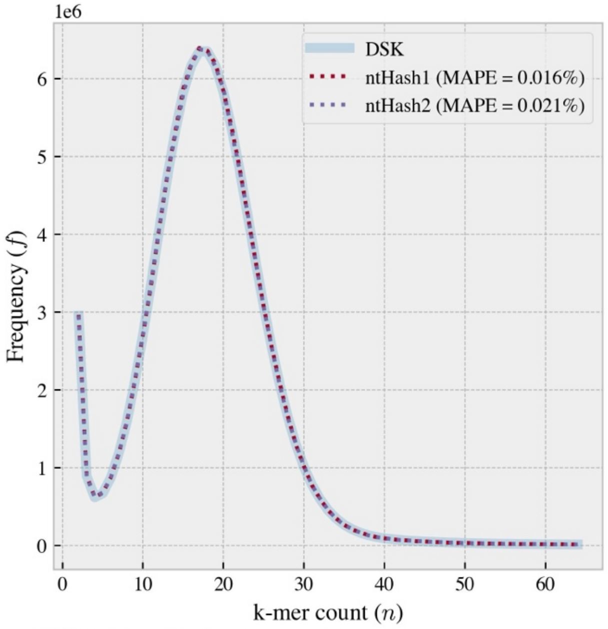

Fig. S9. C. elegans k-mer profiles generated with ntCard using both the previous and new versions of ntHash (k=80). ntCard is also able to estimate k-mer profiles with low error when integrated with ntHash2.

Our final experiment shows an application of spaced seed hashing in long-read data. We demonstrate the effect of using spaced seeds for estimating the coverage of a dataset with ntCard. By increasing the number of base-pairs in the k-mers, the coverage profiles become more sensitive to errors, especially in datasets with longer reads and higher error rates such as the CHM13 dataset[1] with a coverage profile shown in Fig. S10. The data used in this experiment contains approximately 56.8x nominal coverage of the PacBio HiFi data used in the assembly of the telomere-to-telomere human genome (Nurk et al., 2022). Here, we use gapped spaced seeds with lengths of l bases and w care positions. The seeds are built as two equal and contiguous care blocks separated by $\Delta = l - w$ do not care positions. By using gapped spaced seeds with the same lengths and different number of bases included as care positions, the peaks of the profiles remain in roughly the same position. On the other hand, by increasing the number of bases in k-mers, the peak gets shifted to the right. In other words, utilizing spaced seeds for cardinality estimation results in more stable graphs because of the level of error-tolerance they have compared to contiguous k-mers.

[1] Available at https://github.com/marbl/CHM13

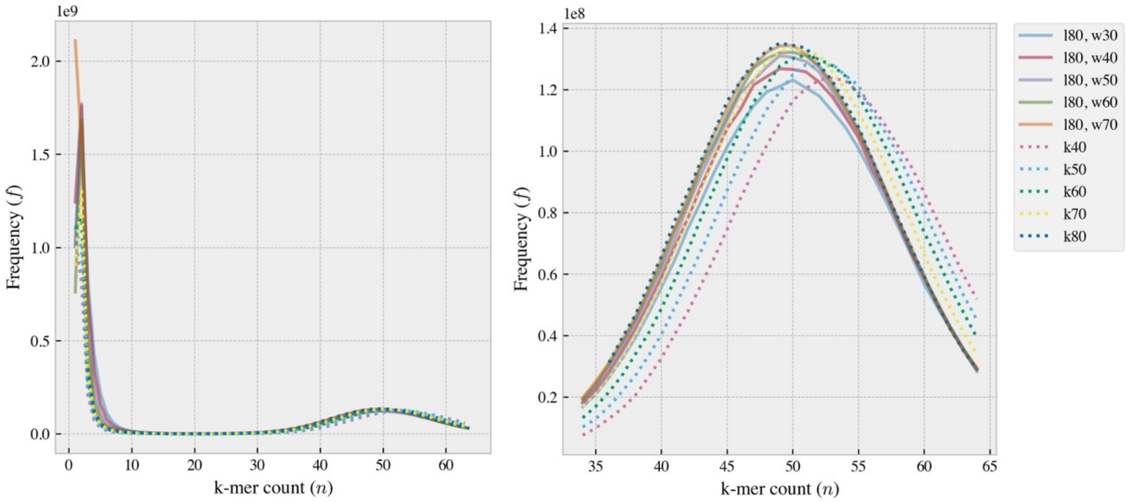

Fig. S10. Dataset coverage estimation using ntCard with k-mers and spaced seeds. The plot on the right is a zoomed-in version of the peak in the count range between 34 and 64. Spaced seeds are more stable compared to k-mers with the same number of bases and are more reliable for coverage estimation in long-read datasets.

References

Birol, Inanc, Hamid Mohamadi, and Justin Chu. 2018. "ntPack: A Software Package for Big Data in Genomics." *IEEE/ACM International Symposium on Big Data Computing (BDC).* IEEE. 41-50.

Gurevich, Alexey, Vladislav Saveliev, Nikolay Vyahhi, and Glenn Tesler. 2013. "QUAST: quality assessment tool for genome assemblies." *Bioinformatics* 29 (8): 1072-1075.

Hahn, Lars, Chris-André Leimeister, Rachid Ounit, Stefano Lonardi, and Burkhard Morgenstern. 2016. "rasbhari: Optimizing Spaced Seeds for Database Searching, Read Mapping and Alignment-Free Sequence Comparison." *PLOS Computational Biology* (Public Library of Science) 12 (10): 1-18.

Jackman, Shaun D., Benjamin P. Vandervalk, Hamid Mohamadi, Justin Chu, Sarah Yeo, S. Austin Hammond, Golnaz Jahesh, et al. 2017. "ABySS 2.0: resource-efficient assembly of large genomes using a Bloom filter." *Genome Research* (Cold Spring Harbor Lab) 27 (5): 768-777.

Johnson, Norman L., Samuel Kotz, and N. Balakrishnan. 1995. *Continuous Univariate Distributions Volume 2, Second edition.* Wiley.

Massey Jr., Frank J. 1951. "The Kolmogorov-Smirnov Test for Goodness of Fit." *Journal of the American Statistical Association* (Taylor & Francis) 46 (253): 68-78.

Mohamadi, Hamid, Hamza Khan, and Inanc Birol. 2017. "ntCard: a streaming algorithm for cardinality estimation in genomics data." *Bioinformatics* 33 (9): 1324–1330.

Mohamadi, Hamid, Justin Chu, Benjamin P. Vandervalk, and Inanc Birol. 2016. "ntHash: recursive nucleotide hashing." *Bioinformatics* 32 (22): 3492-3494.

Nurk, Sergey, Sergey Koren, Arang Rhie, Mikko Rautiainen, Andrey V. Bzikadze, Alla Mikheenko, Mitchell R. Vollger, et al. "The Complete Sequence of a Human Genome." Science 376, no. 6588 (2022): 44–53. https://doi.org/10.1126/science.abj6987.

Ounit, Rachid, and Stefano Lonardi. 2016. "Higher classification sensitivity of short metagenomic reads with CLARK-S." *Bioinformatics* 32 (24): 3823–3825.

Rizk, Guillaume, Dominique Lavenier, and Rayan Chikhi. 2013. "DSK: k-mer counting with very low memory usage." *Bioinformatics* 29 (5): 652–653.

Zook, Justin M., David Catoe, Jennifer McDaniel, Lindsay Vang, Noah Spies, Arend Sidow, Ziming Weng, et al. 2016. "Extensive sequencing of seven human genomes to characterize benchmark reference materials." *Scientific Data* (Nature) 160025.
